# Evaluation of Symmetric Dimethylarginine (SDMA) in Dogs with Acute Pancreatitis

**DOI:** 10.3390/vetsci7020072

**Published:** 2020-06-01

**Authors:** Eleonora Gori, Alessio Pierini, Ilaria Lippi, Valentina Meucci, Francesca Perondi, Veronica Marchetti

**Affiliations:** Department of Veterinary Sciences, University of Pisa, Via Livornese Lato Monte, 56121 Pisa, Italy; eleonora.gori@vet.unipi.it (E.G.); ilaria.lippi@unipi.it (I.L.); valentina.meucci@unipi.it (V.M.); f.perondi87@gmail.com (F.P.); veronica.marchetti@unipi.it (V.M.)

**Keywords:** canine, pancreas, inflammation, kidney injury

## Abstract

Symmetric dimethylarginine (SDMA) is considered an important biomarker of kidney dysfunction. The aims of the study were to evaluate SDMA in dogs with acute pancreatitis (AP) and its relationship with the presence of kidney injury and mortality. A cohort study including fifty-four dogs with AP diagnosed using compatible clinical and laboratory parameters, abnormal SNAP cPL and compatible abdominal ultrasound within 48 h from admission, was conducted. Dogs with history of renal and/or urinary diseases were excluded, along with dogs exposed to nephrotoxic drugs. Serum urea and creatinine and urinary output (UO) were recorded. Acute kidney injury (AKI) was diagnosed and graded using International Renal Interest Society (IRIS) guidelines. SDMA was measured using high performance liquid chromatography. Fifty-four dogs were included and divided in non-AKI (*n* = 37) and AKI dogs (*n* = 17). Twenty-three dogs (14 non-AKI) had SDMA > 15 μg/dL. Median SDMA was higher in AKI dogs than non-AKI dogs (25.7 vs. 13.93 μg/dL; *p* = 0.03). Dogs with normal creatinine (AP and AKI 1 dogs) had SDMA above reference range in 38% and 33% of cases, respectively. In AKI dogs, SDMA and creatinine were positively correlated (*p* = 0.006 *r* = 0.7). SDMA was not significantly different between survivors and non-survivors. Although further studies are warranted, SDMA may be a useful tool in canine AP, as a high SDMA may be related to subclinical kidney impairment.

## 1. Introduction

Acute pancreatitis (AP) in dogs has a variety of clinical presentations, including anorexia, weakness, vomiting, diarrhea, and abdominal pain. The clinical presentation of AP depends on the magnitude of inflammation of pancreatic parenchyma, which is followed by the release of inflammatory mediators, such as reactive oxygen species, reactive nitrogen species, and cytokines, which lead to systemic inflammatory response syndrome (SIRS) [1]. Briefly, during AP, a premature activation of pancreatic enzymes within acinar cells causes acinar cell injury, which leads to migration and activation of inflammatory cells and the release of pro-inflammatory cytokines and other inflammatory mediators [1]. The progression on pancreatic injury to surrounding tissues contributes to systemic complications (e.g., acute kidney injury (AKI)) [1,2]. In particular, circulating activated enzymes and proteases can cause endothelial damage, leading to hypovolemia, hypotension, hypercoagulability, and fibrin deposition in the glomeruli [1,2]. Additionally, this inflammatory process stimulates cytokine release and production of oxygen free radicals, which can contribute to the worsening of AKI [1,2]. The AP-induced kidney injury has been widely studied and highlighted especially in humans [3,4,5] but also in dogs [6,7,8] as a negative prognostic factor. In humans, together with acute lung injury, AKI is the main complication linked to a high morbidity and mortality in AP patients [3,4,5]. The same data have been highlighted also in dogs, where kidney injury was evaluated by three studies [6,7,8]. These studies reported above concluded that AP dogs with azotemia, proteinuria, AKI and/or oligoanuria had a higher mortality rate than dogs without them [6,7,8].

Symmetric dimethylarginine (SDMA) is a sensitive renal biomarker whose concentrations increase earlier than creatinine as the glomerular filtration rate decreases [9,10]. To date in humans and small animals, SDMA is considered an important early biomarker of kidney dysfunction [9,10]. SDMA was developed primarily for the detection of non-azotemic chronic kidney disease (CKD), since it was correlated with the glomerular filtration rate [9]. A more recent study applied it on a population of dogs with AKI and CKD, concluding that SDMA was suitable for identifying dogs affected by AKI or CKD, but could not differentiate between them [10].

Since azotemia and AKI have been identified as negative prognostic factors in canine AP [6,7], SDMA may identify a kidney injury earlier in these patients. Our hypothesis was that SDMA can be an earlier marker of kidney injury compared to creatinine in dogs with AP. For this reason, SDMA may help the clinicians to recognize the presence of an early kidney injury in non-azotemic dogs with AP. The aims of this study were: (1) to evaluate SDMA concentrations in dogs diagnosed with AP, (2) to compare SDMA and creatinine in the detection of kidney injury in dogs with AP, (3) to evaluate the possible relationship between SDMA and mortality, and (4), lastly, to evaluate SDMA trends in survivors.

## 2. Materials and Methods

SDMA concentration was evaluated using exceeding serum samples of hospitalized dogs with AP included in a larger prospective study, conducted at our veterinary teaching hospital between March 2017 and April 2019, for which ethical approval had been obtained (Approval No. 16749/2017). Each dog was hospitalized due to AP and admission (T0) exceeding serum samples for each dog were frozen at −80 °C until analysis, which was performed 25 months after collection at the latest. Concentrations of SDMA are reported to be stable for several years when stored at −70/−80 °C [11]. Afterwards, around 2 weeks (10–20 days) from the discharge, survivors were clinically re-checked at our veterinary teaching hospital, and bloodwork were performed according to the clinicians’ decision case by case, and surplus serum was stored at −80 °C until analysis. Serum SDMA concentrations were measured both on T0 (hospital admission) and at the first re-check (T1). Serum concentration of SDMA was determined by high-performance liquid chromatography with fluorescence detection, as previously described by Teerlink [11]. Blood was centrifuged immediately after collection, and serum was frozen at −80 °C and stored until analysis. For SDMA, the intra- and inter-assay coefficients of variation was <9%. The lower limit of quantification, at a signal-to-noise ratio of 10, was 0.5 μg/dL for SDMA, using a 0.2 mL sample volume [12].

The diagnosis of AP was made if there were: compatible clinical signs (two or more of the following: abdominal pain, diarrhea, vomiting, or anorexia/poor appetite), a compatible abdominal ultrasound (Xario XG, Toshiba, Tokyo, Japan) for AP within 48 h of hospital admission, and an abnormal SNAP cPL test result (Idexx Laboratories, Milan, Italy). Abdominal ultrasound was considered consistent with AP if there was a hypoechoic and enlarged pancreas, with an irregular shape and margins, surrounded by hyperechoic mesentery and/or abdominal effusion [13]. Dogs to be included in the study had to have a full hematobiochemical panel (urea, creatinine, albumin, total proteins, glucose, alanine transaminase, aspartate transaminase, alkaline phosphatase, total bilirubin, g-glutamyltransferase, cholesterol, sodium, potassium, chloride, total calcium, phosphorus, and c-reactive protein), a coagulation profile (prothrombin time, activated partial thromboplastin time, and fibrinogen), urinalysis, and venous blood gas performed as the routine care for dogs presented for AP at presentation. For each dog, data on urea, creatinine, and urinary output (UO), extrapolated from the medical records and routinely evaluated over a 6 h window, were recorded. Acute kidney injury (AKI) was diagnosed and graded, based on the current IRIS consensus [14]. AKI diagnosis was based on the following criteria: rapid onset (<1 week) of clinical signs (depression, vomiting, anorexia, weakness, diarrhea), evaluation of hematobiochemical markers compatible with AKI (increase in creatinine ≥0.3 mg/dL or more within 48 h), and evidence of oliguria and/or anuria. In addition, dogs were subgraded as non-oliguric (>1 mL/kg/h over 6 h) or oligoanuric (<1 mL/kg/h over 6 h) based on the UO [14].

Dogs with a history of both acute and chronic renal disease (clinical records/history, bloodwork, and diagnostic imaging), urinary tract infection and/or on hemodialysis treatment were excluded, along with dogs that had received known nephrotoxic drugs (e.g., non-steroidal anti-inflammatory drugs, aminoglycosides). In our hospital, all dogs with documented AKI, or which developed AKI during hospitalization, are tested for leptospirosis (and considered negative if two MAT tests were negative two weeks apart; or if no sufficient seroconversion (MAT > 1:800) was noticed in vaccinated dogs). As a hospital policy, we test all AKI dogs for leptospirosis due to the high zoonotic risk. Urine culture was available for all dogs with AKI and patients with positive urine culture were excluded from the study. Pyelonephritis was considered unlikely if urine culture was negative. 

Canine acute pancreatitis severity (CAPS), which is a recently validated clinical scoring system for short-term mortality in dogs with AP [15], was calculated for each dog at presentation (T0). The previously described cut-off of 11 was used to divide the dogs into two groups, since it was showed to be the most sensible and specific cut-off for the short-term death (CAPS < and ≥ 11) [15]. The CAPS score was calculated as CAPS score = 8 × (1 if SIRS, 0 otherwise) + 3 × (1 if coagulation disorders, 0 otherwise) + 4 × (1 if increased creatinine, 0 otherwise) + 3 × (1 if ionized hypocalcemia, 0 otherwise) [15]. The presence of a systemic inflammatory response syndrome (SIRS) was assessed using recently proposed criteria [15,16]. A dog was assigned to the SIRS group if at least two of the following four criteria were present: hyperthermia or hypothermia (>39.7 °C or <37.8 °C), tachycardia (>160 beats/minute), tachypnea (>40 breaths/minute), white blood cells (WBC) < 4000/μL, or >12,000/μL or band neutrophils >10% [15,16]. Data about complete blood count, creatinine, and ionized calcium at presentation were recorded in order to calculate the CAPS score. The complete blood count was performed using a laser cell counter (Procyte DX, IDEXX Laboratories, Westbrook, ME, USA) and a blood smear stained with May-Grünwald Giemsa (Aerospray Wescor, Delcon, Milan, Italy), examined by experienced and trained clinical pathologist microscopically. Serum urea and creatinine was evaluated on serum samples using an automatized biochemistry analyzer (Liasys, Assel SRL, Rome, Italy). Ionized calcium was evaluated using a blood gas analyzer (ABL 700 series, Radiometer Medical, Copenhagen, Denmark). The coagulation profile was performed using an automated coagulation analyzer (Destiny Max, Tcoag Ireland Inc., Wicklow, Ireland).

Mortality was evaluated at hospital discharge and dogs were divided into survivors (discharged from the hospital) and non-survivors (died or euthanized for the worsening of the clinical condition despite therapy). 

All continuous parameters (age, weight, urea, creatinine, SDMA) were tested with the Kolmogorov–Smirnov normality test. Normally distributed variables were expressed using the mean ± standard deviation (SD), while the non-normally distributed variables were expressed using the median and interquartile range (IQR). Dogs were divided into groups based on the presence of AKI (AKI and non-AKI dogs) and on CAPS score (CAPS < 11 and CAPS ≥ 11; [15]). SDMA and creatinine were correlated with the Spearman’s correlation test both in AKI dogs and non-AKI dogs. A univariable analysis was performed to identify variables associated with the mortality using *t*-tests or the Mann–Whitney U-test based on the normality distribution (age, weight, urea, creatinine, and SDMA) or Chi-square test or Fisher’s exact test (CAPS score groups, presence of AKI, UO). Finally, T1 SDMA concentrations in survivors were compared to T0 SDMA (Wilcoxon matched pair sign rank test) to establish if, after the remission of AP clinical signs, survivors had undergone a significant reduction of SDMA. Data were analyzed using commercial software (IBM SPSS Statistics, version 25, IBM Corporation, New York, New York; GraphPad Prism, version 7.0 a, GraphPad Software Inc, San Diego, CA, USA) and a *p*-value of <0.05 was considered statistically significant.

## 3. Results

Fifty-four dogs diagnosed with AP were enrolled with the owners’ informed consent. The mean age was 10.4 ± 3.5 years. The breeds described were Poodle (*n* = 4), German Shepherd (*n* = 2), Lagotto Romagnolo (*n* = 2), Labrador Retriever (*n* = 2), Beagle (*n* = 2), Jack Russell terrier (*n* = 2), Springer Spaniel (*n* = 2), Shih Tzu (*n* = 2), and Spitz (*n* = 2). Seventeen dogs (33%) were mixed breed. Twenty-five out of 54 dogs (46.3%) were females, of which eight were spayed, while the other 29 dogs were males (53.7%), of which 12 were neutered. The median weight was 16.5 kg, ranging from 3.3 kg to 40.7 kg. In our cohort of dogs, the CAPS score ranged from 0 to 15. Evaluating the CAPS score: one dog had a CAPS score = 3, 31 dogs had a CAPS score = 8, while five and eight dogs had a CAPS score of 11 and 12, respectively. Only one dog had CAPS score = 15. Seventeen dogs (31.5%) were assigned to the non-survivor group. 

**Figure 1:**
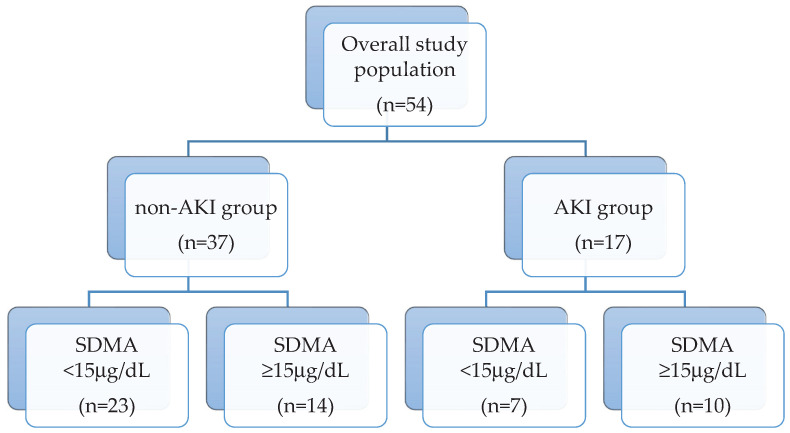
Flow diagram representing the study population groups (non-AKI and AKI) and their serum symmetric dimethylarginine (SDMA) concentration subdivision (SDMA < and ≥15 μg/dL).

The study population groups and their serum SDMA concentration are reported in Figure 1. Overall median SDMA was 13.8 μg/dL (IQR 15.8 μg/dL). Seventeen dogs (31.4%) showed AKI, of which 11 were oligo-anuric. Nine dogs had AKI grade one, three dogs had AKI grade 2, and four dogs and one dog had AKI grades 3 and 4, respectively. 

**Figure 2:**
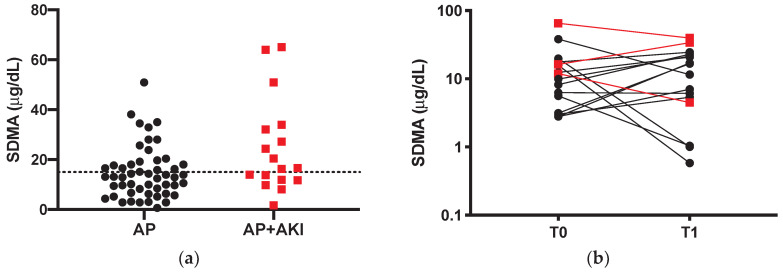
(**a**) SDMA values of non-azotemic dogs with acute pancreatitis (AP; black dots) and in dogs with acute pancreatitis (AP) and acute kidney injury (AKI) (AP + AKI; red squares). The line represents the threshold values of 15 μg/dL. (**b**) Comparison between admission (T0) and first recheck (T1) SDMA of survivors (*n* = 16). A logarithmic (base 10) scale is used for the vertical axis. Black dots represent AP dogs and red squares represent AP + AKI dogs.

Twenty-three dogs (45%), of which 14 were in the non-AKI group, had SDMA above our internally validated reference range (max 15 μg/dL) [12] (Figure 2a). Median SDMA was significantly higher in AKI dogs than non-AKI dogs (25.7 vs. 13.93 μg/dL; *p* = 0.03). Comparisons between T0 SDMA and T0 creatinine of the overall population are displayed in Table 1. Briefly, dogs with normal creatinine (AP and AKI 1 dogs) had SDMA above the reference range in 38% and 33% of the cases, respectively. 

Serum creatinine concentration shows a strong positive correlation with SDMA level in AKI dogs (*p* = 0.006, *r* = 0.7), although the same correlation is not significant in the non-AKI group (*p* = 0.50). Lastly, dogs with CAPS score ≥11 had a higher SDMA level (median 19.5, interquartile range (IQR) 26.3 μg/dL) than dogs with CAPS <11 (median 12.7, IQR 12 μg/dL; *p* = 0.036).

The univariable analysis results are shown in Table 2. Briefly, non-survivors show higher prevalence of AKI and oligoanuria (64.7%) and higher median SDMA concentration (16.6 vs 11.67 μg/dL) than survivors (Table 2).

Sixteen out of 37 serum samples (43.2%) of survivors were available for the SDMA evaluation at the first recheck (Table 3). Only six dogs had a decrease in SDMA level at T1 compared to T0, while eight out of 16 dogs had a T1 SDMA above the reference range, in fact there were no differences in T0–T1 in SDMA levels in survivors (10.92 vs. 14.10 μg/dL; *p* = 0.99). 

## 4. Discussion

To the best of our knowledge, this is the first study assessing SDMA concentration in dogs with naturally occurring AP. In canine AP, based on our results, although only a few dogs showed azotemia, about half of the overall population had an abnormal SDMA level compared to our upper values of the reference interval (15 μg/dL) [12]. 

Unlike humans, SDMA has been widely investigated in dogs. As stated above, although asymmetric dimethylarginine is mainly cleared by enzymatic hydrolysis, SDMA is primarily eliminated by the kidneys, and thus it has been considered as an early marker of kidney disfunction [17,18]. Furthermore, SDMAs seem to have fewer non-renal influences, in terms of age, sex, breed, and lean body mass compared to serum creatinine [18]. Excluding dogs with a known history of renal disease, urinary tract infection and dogs that had received known nephrotoxic drugs, we thus reduced the interpretation biases linked to the abnormal SDMA values potentially pre-existent in non-azotemic dogs.

Interestingly, about half of our dogs show a higher SDMA than the upper limit of our reference range and, comparing SDMA and creatinine, SDMA seems to better highlight kidney impairment, even in non-azotemic dogs (AP and AKI 1; Table 1). We hypothesize that this finding is due to a non-azotemic subclinical kidney injury linked to AP, which SDMA can identify at an early stage. One of the first studies on SDMA on dogs showed how SDMA can detect <30% loss of renal function compared to serum creatinine, which increases when about 50% of the renal function mass is lost [17]. More recently, SDMA has been shown to be a reliable marker of AKI in dogs [10], similarly to previous findings reported in human patients [19]. This study by Dahlem and colleagues found that SDMA is a feasible marker for the detection of renal impairment, although AKI and CKD dogs had similar plasma SDMA concentrations (39.5 vs. 35.0 μg/dL, respectively) [10]. Based on these considerations, renal injury during AP may be frequent, even compared to what is reported in the literature. Previous studies on kidney injury in canine AP showed how about 25% of dogs with AP may have AKI [7] and markers of urinary kidney injury (e.g., proteinuria, casts) may be present in 37% of dogs with AP [8].

As previously reported [10,17,18], in our study SDMA had a significant positive correlation with serum creatinine in AKI dogs, although this did not in the non-AKI group. As hypothesized by Kopke and colleagues [20], in non-azotemic dogs, the relationship between SDMA and serum creatinine may be non-linear.

A significant association between SDMA concentration and CAPS score is also found. Since CAPS score is a recently developed severity score for canine AP, we can hypothesize that dogs with higher CAPS, thus with a more severe disease, may probably have a kidney injury.

We also highlighted a significant association between the presence of AKI, oligoanuria, and the mortality, as well as high SDMA. This finding agrees with a recent paper on AKI and AP in dogs, in which both AKI and oligoanuria were associated with the outcome [7]. As in humans, an AP-induced kidney impairment, as it may be also part of a multiorgan dysfunction syndrome [1,2,3,4,5], can influence negatively patients’ prognosis. 

Lastly, evaluating the SDMA after recovery from AP, we fail to find a significant reduction of SDMA between T0 and T1, in fact, as reported in Figure 2b, many dogs had rather an increase in SDMA. This particular finding is not easy to discuss, since there are no available data about the kinetics of SDMA either in AP dogs, or in dogs with AKI. The lack of decrease in SDMA may be due to the timing of the first recheck (10–20 days), which can be too short for a complete kidney injury recovery, or due to a persistent subclinical kidney injury that can also progress to chronic kidney injury. Furthermore, the last proposed hypothesis gives to clinicians the useful advice to monitor, and possibly manage with specific treatments, dogs that do not fully recover in order to slow the kidney injury progression. 

This study has several limitations. Firstly, a recent study showed a subjective mild variability in SDMA concentration in apparently healthy dogs [19], thus we cannot rule out that abnormal SDMA concentration may be the result of a subjective variability. In the follow-up analysis, SDMA evaluation was available only for 16 dogs in the survivor group, and, thus, it would be interesting to have a wider study population to test our results. Secondly, naturally occurring canine AP is very often accompanied/caused by other comorbidities, such as chronic enteropathies and endocrinopathies. However, such comorbidities cannot be ruled out, and they were not taken into account in evaluating the mortality. Lastly, other urinary and/or serum early markers of AKI (e.g., neutrophil gelatinase-associated lipocalin, fractional excretion of electrolytes) have not been investigated in our population. AKI dogs were not divided into volume-responsive or intrinsic groups. As volume-responsive AKI has been showed to have a better prognosis than intrinsic AKI [21], a further investigation of these two groups would be interesting.

## 5. Conclusions

Our study revealed that SDMA is higher in AKI dogs with AP and it may be useful in the detection of non-azotemic dogs, as AKI grade 1. Although further larger-scale prospective studies are needed, interestingly, about a third of non-AKI dogs with AP presented abnormal SDMA, which may be related to subclinical kidney impairment. 

## Figures and Tables

**Table 1:** Comparison between SDMA and serum creatinine (sCr) values in AP and AP + AKI dogs, divided into AKI grades (1–4).

T0 (*n* = 54)				
Group	SDMA (μg/dL) ^1^	SDMA > 15 ^2^	sCr (mg/dL)	sCr > 1.5 ^2^
AP (*n* = 37)	12.4 (12.3)	14 (38%)	0.9 (0.4)	0 (0%)
AP + AKI 1 (*n* = 9)	13.7 (13.4)	3 (33%)	1.1 (0.9)	0 (0%)
AP + AKI 2 (*n* = 3)	16.6 (16.2–50.9) ^3^	3 (100%)	2.2 (1.7–2.5) ^3^	3 (100%)
AP + AKI 3 (*n* = 4)	48.9 (47.5)	3 (75%)	3.6 (1.2)	4 (100%)
AP + AKI 4 (*n* = 1)	32	1 (100%)	8.9	1 (100%)

^1^ Data are displayed as median and interquartile range (IQR) in brackets ^2^ Data are displayed as absolute and relative frequency. ^3^ The IQR was not calculated due to the small number of dogs in this group, the values in brackets range from min to max.

**Table 2:** Results from the univariable analysis of selected variables between survivors (*n* = 37 dogs) and non-survivors (*n* = 17).

Variable	Survivors (*n* = 37)	Non-Survivors (*n* = 17)	*p*-Value
Age (years)	10.3 ± 3.7	10.6 ± 3.1	0.83
Weight (kg)	14.4 (19.3)	23.5 (16.5)	0.27
CAPS ≥ 11	6/37	8/17	0.01
Urea (mg/dL)	41 (49.5)	68 (80.5)	0.19
Creatinine (mg/dL)	1 (0.5)	1 (1)	0.35
Presence of AKI	5/37	11/17	<0.0001
Presence of oligoanuria	0/37	11/17	<0.0001
SDMA (μg/dL)	11.7 (12.1)	16.6 (17.2)	0.019

Normally distributed variables are expressed using mean ± standard deviation (unpaired *t*-test), while the non-normally distributed variables are expressed using median and interquartile range (IQR) in brackets (Mann-Whitney U-test). Abbreviations: AKI, acute kidney injury; CAPS, canine acute pancreatitis severity; SDMA, symmetric dimethylarginine. The variables in bold are those that resulted statistically significant.

**Table 3:** Evaluation of first recheck (T1) SDMA and serum creatinine (sCr) values in AP and AP + AKI dogs, divided into AKI grades (1–4).

T1 (*n* = 16)		
Group	SDMA (μg/dL) ^1^	sCr (mg/dL) ^1^
AP (*n* = 13)	11.5 (17.9)	0.8 (0.35)
AP + AKI 1 (*n* = 1)	4.5	0.7
AP + AKI 2 (*n* = 1)	33.9	0.6
AP + AKI 3 (*n* = 1)	39.8	1.9
AP + AKI 4 (*n* = 0)	---	---

^1^ Data are displayed as median and IQR in brackets.
